# Influence of Physical Exercise on Advanced Glycation End Products Levels in Patients Living With the Human Immunodeficiency Virus

**DOI:** 10.3389/fphys.2018.01641

**Published:** 2018-12-06

**Authors:** Karine Lino Rodrigues, Juliana Pereira Borges, Gabriella de Oliveira Lopes, Evelyn Nunes Goulart da Silva Pereira, Mauro Felippe Felix Mediano, Paulo Farinatti, Eduardo Tibiriça, Anissa Daliry

**Affiliations:** ^1^Laboratory of Cardiovascular Investigation, Oswaldo Cruz Institute, Oswaldo Cruz Foundation, Rio de Janeiro, Brazil; ^2^Laboratory of Physical Activity and Health Promotion, State University of Rio de Janeiro, Rio de Janeiro, Brazil; ^3^Evandro Chagas National Institute of Infectious Diseases, Oswaldo Cruz Foundation, Rio de Janeiro, Brazil; ^4^National Institute of Cardiology, Ministry of Health, Rio de Janeiro, Brazil

**Keywords:** combined antiretroviral therapy, HIV, cardiovascular diseases, advanced glycation and products, physical training

## Abstract

**Introduction:** Combined antiretroviral therapy (cART) used to treat acquired immunodeficiency virus (HIV) induces a number of adverse effects, such as insulin resistance and dyslipidemia, which ultimately increases the cardiovascular risk. Advanced glycation end products (AGEs) have been implicated in the etiology of cardiovascular diseases, diabetes and other chronic diseases. It is known that physical exercise improves the lipid profile, insulin resistance and reduces the risk of cardiovascular diseases. However, the impact of physical exercise on AGE levels in HIV-infected patients has not been so far investigated. Therefore, this study compared AGEs levels in people with and without HIV and verified the effect of physical training on serum AGE levels.

**Methods:** Participants were initially assigned into three groups: healthy control (CTL, *n* = 35), physically inactive HIV-infected (In-HIV, *n* = 33) and physically active HIV-infected (Ac-HIV, *n* = 19). The In-HIV group underwent physical training for 3 months, consisting of 60-min sessions of multimodal supervised exercise (aerobic, resistance and flexibility) with moderate intensity (50–80% heart rate reserve), performed 3 times/week. AGEs were measured in serum by fluorescence spectrometry.

**Results:** At baseline, serum AGEs fluorescence level was significantly higher in inactive HIV-patients when compared to controls or active HIV-patients (In-HIV: 0.93 ± 0.08 vs. controls: 0.68 ± 0.13 and Ac-HIV: 0.59 ± 0.04 A.U.; *P* < 0.001). Triglycerides were also higher in In-HIV than CTL (182.8 ± 102 vs. 132.8 ± 52.3 mg/dL; *P* < 0.05). Waist circumference was lower in Ac-HIV, compared to In-HIV and controls (83.9 ± 10.4 vs. 92.9 ± 13.5 and 98.3 ± 12.4, respectively; *P* < 0.05). Body mass, fasting blood glucose, LDL, HDL, and total cholesterol were similar between groups. After training, AGE levels decreased (Baseline: 0.93 ± 0.08 vs. 3 months follow-up: 0.59 ± 0.04 AU; *P* < 0.001), no further difference being detected vs. CTL or Ac-HIV. Conclusion: HIV-infected patients under cART exhibited elevated AGEs levels compared to healthy individuals and physically active patients. Short-term aerobic training of moderate intensity counteracted this condition.

## Introduction

The clinical benefits of cART for treating HIV infection are well established. However, several metabolic complications have been associated to continuous cART, such as insulin resistance and dyslipidemia, which contribute to atherosclerosis in people living with HIV (PLWH) (Freiberg et al., 2013; Drozd et al., 2017). Indeed, CVDs including CAD, has been shown to be the main cause of premature morbidity and mortality in PLWH under cART (Currier et al., 2008; Freiberg et al., 2013; Silverberg et al., 2014).

Advanced glycation end products are proteins or lipids that become glycated as a result of exposure to reduced sugars (Singh et al., 2001). These products may increase the risk of development, or worsening, of a number of degenerative diseases, such as diabetes, CVD, chronic kidney and liver diseases, and neurodegenerative syndromes, such as Alzheimer disease (Genuth et al., 2005; Hyogo et al., 2007; Kerkeni et al., 2014; Silvares et al., 2016; Pereira et al., 2017). AGEs damage tissues and cells in three major ways: (a) direct modification of intracellular proteins; (b) modifications of extracellular matrix proteins, resulting in abnormal interactions between these proteins and cells; (c) receptor-mediated production of reactive oxygen species with activation of transcription nuclear factor-κB leading to deleterious changes in cellular processes (Goldin et al., 2006; Toure et al., 2008). Furthermore, the binding of AGEs to their RAGE receptor promotes the activation of proinflammatory and procoagulant cellular pathways that increases the production of adhesion molecules, cytokines such as IL-6 and TNF-α, and decreases the bioavailability of NO (Goldin et al., 2006).

Few studies have so far investigated the role of AGEs in HIV infection and they showed controversial results. Recently, Sprenger et al. (2017) demonstrated that HIV-1 patients had increased accumulation of AGEs in the skin in comparison with healthy individuals, while Tasca et al. (2016) showed decreased AGE levels after 6 months of cART introduction. Therefore studies which aim to address the participation of AGE in physiopathology of HIV-infection and cART treatment are necessary to dissect the mechanisms involved in metabolic complications associated to continuous cART.

On the other hand, evidence from animal studies has demonstrated that exercise reduces the concentration of AGEs and highly reactive intermediates of AGE pathway, such as methylglyoxal (Gu et al., 2014a,b). Although it is already clear that exercise training improves insulin resistance and lipid profile in PLWH (Grace et al., 2015), there is a lack of data on the effect of exercise training in serum AGEs levels of these patients. Thus, we compared the AGEs levels in healthy individuals vs. physically active and inactive PLWHA under cART. Additionally, we investigated the impact of exercise training in plasma AGEs levels in PLWH. We tested the hypothesis that HIV-infected patients under supervised exercise training have lower AGEs levels compared to sedentary controls. As a consequence of AGE modulation by exercise training in HIV-infected patients those subjects could have a better cardiovascular health profile.

## Materials and Methods

### Study Population and Design

In a cross sectional design, we enrolled 52 HIV-infected patients followed at a tertiary hospital in Rio de Janeiro, Brazil, and 35 healthy control individuals randomly recruited from the staff of the National Institute of Cardiology (Rio de Janeiro, Brazil) matched for age. The HIV-infected patients were assigned into a physically active group (Ac-HIV) and into a physically inactive group (In-HIV), according to their physical activity levels. The following exclusion criteria were considered: (a) cerebral toxoplasmosis or any infectious diseases compromising the central nervous system; (b) use of anabolic hormone therapy or ergogenic resources to gain muscle mass; and (c) cardiovascular, respiratory, bone, muscle, or joint problems that could limit physical function.

After baseline assessments, a subsample of the inactive group engaged in the multimodal supervised exercise program and participants were retested after 3 months of exercise. The primary outcome considered in the present study was AGE levels. Secondary outcomes included: body weight, fasting blood glucose, total cholesterol, LDL-cholesterol, HDL-cholesterol, triglycerides, lipids ratio and waist circumference.

All volunteers provided informed written consent before participation in the study. The present study complied with recommendations of Helsinki Declaration and gained approval from the Research Ethics Committee of the National Institute of Cardiology (protocol #CAAE 42162815.5.0000.5272). The protocol of the study was registered and made public on ClinicalTrials.gov (identifier NCT03343522).

### Multimodal Supervised Exercise Program

The supervised exercise training consisted of 3 months of multimodal exercise (60-min sessions, three times per week), including aerobic, resistance and flexibility exercises, as described elsewhere (Farinatti et al., 2010; Paes Lda et al., 2015; Lopes et al., 2018). Briefly, continuous aerobic training was performed on cycle ergometer or treadmill for 30–40 min at moderate intensity (50–80% reserve heart rate) and resistance training included 8–10 exercises (free weights and machines) performed with 2–3 sets of 12–15 repetitions using 40–60 s of rest interval between sets and load corresponding to 80–90% of 12 repetition maximum. Load progression was performed through a 12 repetition maximum test applied every 2 weeks.

### Anthropometric Assessment

Anthropometric evaluation included measurements of height and body mass. BMI was computed as body mass in kilograms divided by the square of height in meters (Kg/m^2^). Height was measured by means of a wall-mounted stadiometer, and body mass using a calibrated digital scale. Height and body mass were recorded to the closest 0.01 m and 0.1 kg, respectively (Borges et al., 2016).

### Plasma AGEs and Biochemical Assessment

Fasting blood samples were collected and specimens were immediately centrifuged and stored at -70°C until tested. For AGEs evaluation, the fluorescence intensity of serum samples (diluted 1:200 in PBS) was measured at 445 nm after excitation at 370 nm, on a SpectraMax M5 ELISA Microplate Reader (Molecular Devices^TM^, Acton, MA, United States) operating at room temperature. A solution of BSA (1 mg/mL in 0.1 N NaOH) was used as reference and its fluorescence intensity defined as 1U fluorescence. The amount of fluorescence of the patient serum sample was measured at a protein concentration of 1 mg/mL and expressed in arbitrary units (AU) after normalization with the BSA solution. Glucose, total cholesterol, HDL cholesterol, and triglycerides were determined by means of a photometric colorimetric optical system (Cobas Mira^TM^ systems, Roche Diagnostic Corporation, Indianapolis, IN, United States). The LDL cholesterol fraction was calculated by the Friedewald’s formula.

### Statistical Analysis

Sample size was calculated to detect AGEs differences between healthy individuals versus physically active and inactive PLWH under cART. Considering a between-group difference of 0.62 nmol/mL for plasma AGEs levels, with a standard 132 deviation of 0.57 nmol/mL (Macías-Cervantes et al., 2015) and assuming 80% of power with 5% significance level, a minimum of 15 patients in each group was necessary. The estimation was performed using GPowerTM 130 software (version 3.0.10, University of Kiel, Kiel, Germany).

Results are presented as mean ± standard deviation (continuous variables) or percentages (categorical). Shapiro-Wilk test demonstrated a non-normal distribution for AGEs levels, and therefore these data are expressed as median (interquartile range). For the cross-sectional analysis, comparison between groups was performed by means of one-way ANOVA (continuous) or chi-squared test (categorical), except for AGEs levels that was compared using the Kruskal-Wallis test followed by Dunn tests as post hoc verifications.

A per-protocol analysis to evaluate the differences between AGEs levels before and after exercise training in a subsample of inactive group of HIV-patients was tested using linear mixed models. Residual plots of longitudinal analysis were visually inspected and did not demonstrate substantial deviations from the regression assumptions. Differences with a value of *P* ≤ 0.05 were considered as statistically significant. In all cases, calculations were made using the Stata 13.0 software (College Station, Texas, United States).

## Results

**FIGURE 1:**
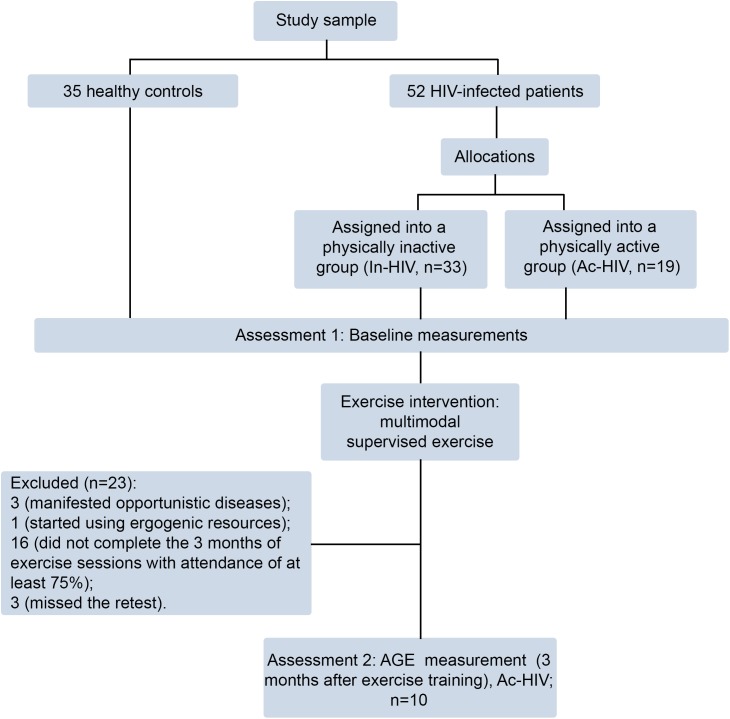
Flow chart of the study design.

First, from the 52 HIV-infected patients, 19 were assigned into a physically active group (Ac-HIV) and 33 into a physically inactive group (In-HIV), according to their physical activity levels. Active patients participated for at least 1 year (mean 5.6 ± 2.9, range 1–10 years) in a multimodal supervised exercise program, while inactive and healthy control individuals reported not having performed any regular physical activity during the same period (Figure 1). The baseline characteristics of the patients are shown in Table 1. Body mass index was lower in Ac-HIV patients (*P* < 0.01) and triglycerides were higher in In-HIV patients (*P* < 0.05) compared to controls, while waist circumference were lower in Ac-HIV compared to In-HIV patients and controls (Table 1). Additionally, protease inhibitors were more frequently used as medication in Ac-HIV than In-HIV patients (*P* ≤ 0.05). The other analyzed variables were similar between groups (Table 1).

**Table 1:** Clinical and biological parameters of Control subjects, Inactive HIV-infected Patients (In-HIV) and Active HIV-infected Patients (Ac-HIV).

	Controls (*n* = 35)	In-HIV (*n* = 33)	Ac-HIV (*n* = 19)
Age (years)	59.4 ± 9.4	49.6 ± 6.2	53.1 ± 6.1
Male (*n*, %)	12 (35%)	21 (63.6%)	14 (73.7%)
Years diagnosed with HIV	**-**	18.5 ± 5.6	18.4 ± 5.9
Years taking cART	**-**	15.2 ± 5.5	14.7 ± 6.4
T CD4 (cell/mm^-3^)	**-**	610.6 ± 211.1	603.2 ± 246.0
Body weight (kg)	73.6 ± 14.9	72.5 ± 14.6	69.2 ± 15.1
Body mass index (kg/m^2^)	28.2 ± 4.8	25.7 ± 5.6	23.4 ± 3.5^∗∗^
Fasting blood glucose (mg/dL)	95.3 ± 13.7	104.4 ± 38.8	93.6 ± 7.5
Total cholesterol (mg/dL)	183.7 ± 34.0	183.5 ± 39.4	167.6 ± 35.8
LDL-cholesterol (mg/dL)	115.9 ± 29.2	106.8 ± 32.8	99.2 ± 29.9
HDL-cholesterol (mg/dL)	41.1 ± 10.8	41.0 ± 11.6	39.9 ± 11.0
Triglycerides (mg/dL)	132.8 ± 52.3	182.8 ± 102.1^∗^	139.1 ± 62.7
Lipids ratio			
LDL/HDL	2.9 ± 1.0	2.7 ± 0.8	2.5 ± 0.6
TGL/HDL	3.8 ± 3.4	5.1 ± 4	3.8 ± 2.2
Waist circumference	98.3 ± 12.4	92.9 ± 13.5	83.9 ± 10.4^∗∗,#^
cART medication (n,%)			
Nucleoside reverse transcriptase inhibitors	**-**	30 (90)	15 (79)
Non- Nucleoside reverse transcriptase inhibitors	**-**	15 (45)	8 (42)
Protease inhibitors	**-**	28 (84)	11 (58)^#^
Integrase inhibitors	**-**	3 (9)	2 (10)
Metabolic syndrome	**-**	5 (15)	0 (0)
Family history of stroke and/or cardiovascular diseases^+^	**-**	27 (82)	15 (79)


*Data represent means ± SD. ^∗^P < 0.05 vs. Controls; ^∗∗^P ≤ 0.01 vs. Controls. ^#^P ≤ 0.05 vs. In-HIV. cART: combination antiretroviral therapy. ^+^Cardiovascular diseases includes: CAD, diabetes, hypertension, IAM.*

**FIGURE 2:**
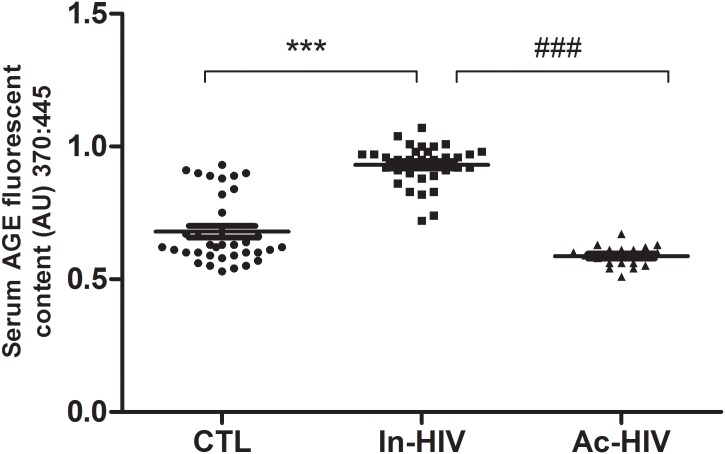
Serum AGE levels of Control Healthy Individuals (CTL, *n* = 35), Inactive HIV-patients (In-HIV, *n* = 33) and Active HIV-patients (Ac-HIV, *n* = 19). Data are presented as median (interquartile range). ^∗∗∗^*P* < 0.001, ^###^*P* < 0.001 between groups.

As exhibited in Figure 2, serum AGEs fluorescence level at baseline was significantly higher in Inactive HIV-patients when compared to Controls and Active HIV-patients (Inactive-HIV: 0.93 ± 0.08 vs. Controls: 0.68 ± 0.13 and Active-HIV: 0.59 ± 0.04 AU; *P* < 0.001) (Figure 2).

**FIGURE 3:**
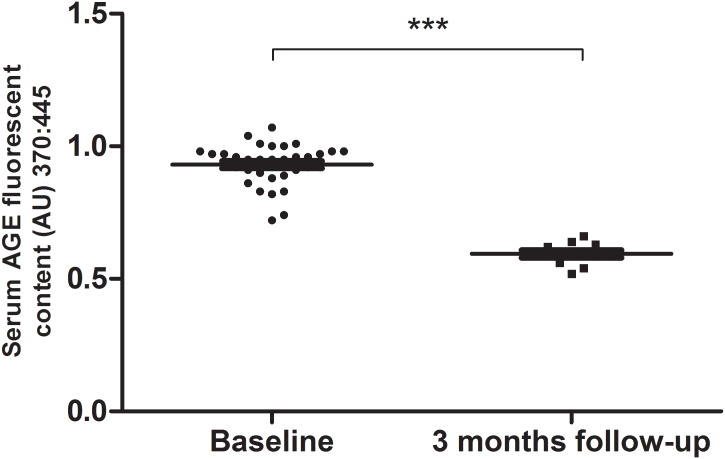
Serum AGEs levels before (baseline, *n* = 33) and after exercise training (3 months follow-up, *n* = 10) of Inactive HIV-infected Patients. Data are presented as median (interquartile range). ^∗∗∗^*P* < 0.001.

After baseline assessments, the inactive group engaged in the multimodal supervised exercise program and participants were retested after 3 months of exercise. Of the initial 33 HIV-patients assigned to the inactive group, 3 were excluded because they have manifested opportunistic diseases, 1 started using ergogenic resources, 16 did not complete the 3 months of exercise sessions with attendance of at least 75% and 3 missed the retest. Therefore, a total of 10 patients of the inactive group were included in the follow-up test after the 3-month supervised exercise training (Figure 1). The serum AGE concentration of In-HIV significantly decreased at 3 months of exercise training follow-up in comparison with baseline (0.93 ± 0.08 vs. 0.59 ± 0.04 AU; *P* < 0.001; Figure 3).

## Discussion

The main findings of the present study were: (1) Inactive HIV-infected patients under cART exhibited higher AGEs and triglycerides serum levels vs. active HIV-infected patients and healthy individuals of similar age and (2) A relatively short multimodal exercise training performed with moderate intensity was able of restoring AGEs concentrations in inactive HIV-infected patients. To our knowledge, this is the first evidence associating AGEs to exercise training in HIV-infected individuals.

Few studies have so far investigated the role of AGEs in HIV infection. Recently, Sprenger et al. (2017), in accordance with our findings, demonstrated in a single-center prospective cohort study that HIV-1 patients had increased accumulation of tissue AGEs in the skin in comparison with healthy age-matched controls, which could represent an independent predictor of CVD in these patients over a 5-year follow-up period (Sprenger et al., 2017). On the other hand, Tasca et al. (2016) showed that after 6 months of the introduction of cART, the plasma levels of AGEs in PLWH decreased. This discrepant result from ours could be due to the shorter period that patients were under cART (6–12 months vs. ∼ 15 years in our study), suggesting that prolonged cART might play a key role in circulating AGEs increasing. It is well accepted that HIV infection causes a persistent low-grade inflammation and immune activation, which increase oxidative stress and contribute to atherosclerosis and other CVD (Hulgan et al., 2003; Grinspoon and Carr, 2005; Blumer et al., 2008; Hsue et al., 2012). Higher AGEs levels might contribute to the worsening of those factors, as they can contribute to comorbidities not related to AIDS, including CVD and diabetes mellitus (Sprenger et al., 2017). Further studies are necessary in order to conclude a causal association between AGE levels increase and CVD and other metabolic disorders in patients infected with HIV under cART, and therefore include as clinical practice, the AGE monitoring in those patients.

Advanced glycation end products can bind to receptors on endothelial cells and activate an intracellular cascade that result in the upregulation of genes related to oxidative stress, inflammation, thrombosis and leukocyte recruitment (de Vos et al., 2014). These factors are acknowledged to increase the risk of endothelial dysfunction and chronic diseases (Singh et al., 2001). A capillary rarefaction and reduced bioavailability of NO triggered by oxidative stress have been suggested as potential causes of endothelial dysfunction in HIV-infected patients (Guzik et al., 2000; Schachinger et al., 2000). We had previously demonstrated that HIV-infected patients exhibit lower arterial compliance and microvascular reactivity in response to pharmacological stimuli in comparison with healthy individuals, suggesting that the combination of HIV infection and cART may lead to endothelial dysfunction (Borges et al., 2011). The formation of atherosclerotic plaques in these patients has been, at least in part, attributed to increased oxidative stress, which could be triggered by elevated circulating and tissue AGEs (Singh et al., 2001; de Vos et al., 2014). The fact that oxidative stress, activation of NADPH oxidase, and decoupling of eNOS play an important role in the development of endothelial dysfunction in individuals with high AGEs levels (as diabetic patients) (Guzik et al., 2000) only reinforce this premise.

Accumulated evidence in the literature demonstrate that exercise training improves endothelial function, not only in healthy individuals, but also in those with increased cardiovascular risk, as the elderly, obese patients, postmenopausal women, or patients with dyslipidemia (Higashi and Yoshizumi, 2004; Barton, 2013). Physical exercise has been recommended as a complementary therapeutic modality for individuals living with HIV/AIDS, however the optimal exercise program (mode, duration, frequency and intensity) that should be prescribed depending on the clinical stage of the HIV-infection are still a matter of debate (Grace et al., 2015). Although exercise training does not confer effects in reducing viral load, it promotes improvements in muscle function, lipid profile, body composition, as well as cART-induced metabolic complications, thus contributing to delay the disease progression (Stanley et al., 2012; Grace et al., 2015; O’Brien et al., 2017). Our findings broaden the current knowledge, as we showed that regular exercise in HIV-infected patients is capable of maintaining the serum AGE levels lower than inactive HIV-infected patients. Moreover, we demonstrated that a combination of resistive and aerobic exercise three times per week for at least 3 months can lead to improvements in AGE outcomes for adults with HIV. Recent experimental studies with animals indicate lowered AGEs levels due to exercise training, particularly in diabetic animals. Ito et al. (2015), for instance, have demonstrated that an 8-week exercise program was capable of reducing AGEs precursors in the kidney of Zucker diabetic rats, thereby lowering the progression of nephropathy. Delbin et al. (2012) also applied 8 weeks of aerobic training to rats with type-1 diabetes reporting a reduction of circulating levels of a specific AGE (N-epsilon-Carboxymethyl-Lysine) in their coronary and femoral arteries, as well as a significant improvement in the vasodilation response to acetylcholine. Finally, Gu et al. (2014a) examined the aorta of older rats submitted to 12 weeks of exercise training and observed that the concentration of highly reactive intermediates of AGE formation, the expression of RAGE, oxidative stress, and inflammation markers were all reduced. Although most research indicating the benefits of exercise training in AGEs has used animal models, exercise-related reduction in circulating AGEs levels in humans has also been demonstrated (Goon et al., 2009; Yoshikawa et al., 2009; Maessen et al., 2017). The decrease in AGE levels has been associated with improvements in several diseases outcomes, therefore, clinicians should consider AGE modulation when prescribing exercise for HIV-infected patients under cART, and recommend exercise programs that provide the wider range of beneficial effects for this population.

To our knowledge no prior study reported the effect of exercise training in AGE levels of HIV-infected individuals. Since AGEs are involved in the pathophysiology of several CVDs, our study adds to the current knowledge by helping to elucidate some of the molecular mechanisms underlying the cardiovascular benefits of regular exercise in this particular group of patients. Our promising findings warrant further research to clarify the role of AGEs within the pathophysiology of CVD in HIV-infected patients under cART, as well as the extent to which different modalities of exercise may counteract this deleterious effect.

### Strengths and Limitations of the Study

The major strength of the study is that it shows that regular exercise or a short period of multimodal exercise training has influence in AGE levels in HIV-infected patients. To our knowledge no prior study reported the effect of exercise training in AGE levels of HIV-infected individuals. The major limitation of this study refers to the higher percentage of men in the Ac-HIV infected group compared to controls. Although there is no difference in AGE levels between men and women reported to date in literature, we cannot assure whether or not the gender has influenced our results. Additionally, the lack of an HIV-infected group not treated with cART precluded further analysis on changes in AGE levels exclusively due to HIV infection. The lack of a group that did not perform exercise intervention was also a study limitation.

### Future Directions

To better understand the role of AGE in the pathogenesis of CVD in HIV-infected patients under cART, the major AGE subtype present in this population should be further evaluated. Moreover, further research on the relation between AGE levels and cardiovascular events in this cohort is warranted.

## Conclusion

In conclusion, AGEs levels were high in HIV-infected patients under cART *vs.* physically active patients and healthy controls with similar age. Moreover, a relative short multimodal exercise intervention (3 months) was capable of restoring AGEs levels in previously inactive HIV-infected patients, approaching their values to those exhibited by healthy or physically active groups. These findings suggest that exercise training might be an effective non-pharmacological intervention to reduce AGEs levels in HIV-infected patients under cART. Further studies are underway to verify if the reduction of AGE levels by exercise training provide a better prognosis for PLWH, including a significant decrease of cardiovascular outcomes, oxidative stress and endothelial dysfunction.

## Ethics Statement

This study was carried out in accordance with the recommendations of Helsinki Declaration with written informed consent from all subjects. All subjects gave written informed consent in accordance with the Declaration of Helsinki. The protocol was approved by the local Institutional Review Board (number CAAE 42162815.5.0000.5272). The protocol of the study was registered and made public on ClinicalTrials.gov (identifier NCT03343522).

## Author Contributions

KR, GL, and EP performed the experiments. KR, JB, MM, and AD analyzed the data. PF, ET, and AD contributed the materials and analysis tools. KR, JB, and AD wrote the paper. KR, JB, MM, PF, ET, and AD revised the article and approved the final version to be published.

## Conflict of Interest Statement

The authors declare that the research was conducted in the absence of any commercial or financial relationships that could be construed as a potential conflict of interest.

## Abbreviations

AGEs: advanced glycation end products
BMI: body mass index
CAD: coronary artery disease
cART: combined antiretroviral therapy
CVD: cardiovascular disease
eNOS: endothelial nitric oxide synthase
HIV: immunodeficiency virus
IL-6: interleukin 6
NADPH: nicotinamide adenine dinucleotide phosphate and nicotinamide
NF-KB: nuclear factor kappa B
NO: nitric oxide
PLWH: people living with immunodeficiency virus
RAGE: receptor for advanced glycation end products
TNF: α- Tumor Necrosis Factor –Alpha
